# G protein‐coupled receptors not currently in the spotlight: free fatty acid receptor 2 and GPR35

**DOI:** 10.1111/bph.14042

**Published:** 2017-11-02

**Authors:** Graeme Milligan

**Affiliations:** ^1^ Centre for Translational Pharmacology, Institute of Molecular, Cell and Systems Biology, College of Medical, Veterinary and Life Sciences University of Glasgow Glasgow G12 8QQ UK

## Abstract

It is widely appreciated that G protein‐coupled receptors have been the most successfully exploited class of targets for the development of small molecule medicines. Despite this, to date, less than 15% of the non‐olfactory G protein‐coupled receptors in the human genome are the targets of a clinically used medicine. In many cases, this is likely to reflect a lack of understanding of the basic underpinning biology of many G protein‐coupled receptors that are not currently in the spotlight, as well as a paucity of pharmacological tool compounds and appropriate animal models to test *in vivo* function of such G protein‐coupled receptors in both normal physiology and in the context of disease. ‘Open Innovation’ arrangements, in which pharmaceutical companies and public–private partnerships provide wider access to tool compounds identified from ligand screening programmes, alongside enhanced medicinal chemistry support to convert such screening ‘hits’ into useful ‘tool’ compounds will provide important routes to improved understanding. However, in parallel, novel approaches to define and fully appreciate the selectivity and mode of action of such tool compounds, as well as better understanding of potential species orthologue variability in the pharmacology and/or signalling profile of a wide range of currently poorly understood and understudied G protein‐coupled receptors, will be vital to fully exploit the therapeutic potential of this large target class. I consider these themes using as exemplars two G protein‐coupled receptors, free fatty acid receptor 2 and GPR35.

**Abbreviations**
4‐CMTB: (S)‐2‐(4‐chlorophenyl)‐3‐methyl‐N‐(thiazol‐2‐yl)butanamide
AZ1729: *N*‐[3‐(2‐carbamimidamido‐4‐methyl‐1,3‐thiazol‐5‐yl)phenyl]‐4‐fluorobenzamide
CATPB: (*S*)‐3‐(2‐(3‐chlorophenyl)acetamido)‐4‐(4‐(trifluoromethyl)phenyl) butanoic acid
DREADD: designer receptor exclusively activated by designer drug
GLPG0974: 4‐[[1‐(benzo[*b*]thiophene‐3‐carbonyl)‐2‐methylazetidine‐2‐carbonyl]‐(3‐chlorobenzyl)amino]butyric acid
SCFA: short‐chain fatty acid

## Introduction

Introductory remarks in many publications and grant applications that centre on aspects of the pharmacology, function or therapeutic potential of members of the http://www.guidetopharmacology.org/GRAC/FamilyDisplayForward?familyId=694&familyType=GPCR superfamily highlight both the historical and ongoing success of targeting GPCRs for the development of medicines to treat human diseases. This undoubtedly reflects the very broad range of physiological end points controlled by GPCR activity and the relative ease of targeting small molecule (low MW) drugs to interact with proteins located predominantly at the external surface of cells. The central roles that neurotransmitters such as noradrenaline, 5‐HT and dopamine play in, for example, cardiovascular and neuronal functions, and the potential for dysregulation of these systems in highly prevalent disease states have resulted in a plethora of medicines targeting GPCRs that are activated by such biogenic amines and recognition of the life‐changing benefits such drugs produce. However, many other GPCRs have not been studied in anywhere near the level of detail as those that respond to biogenic amines, and only in the region of 15% of the non‐olfactory GPCRs are the target of one or more clinically approved small molecule medicine. There are many reasons for this. With more than 350 non‐olfactory GPCRs in the human genome (Alexander *et al.,* 2015a) it is clear that a substantial number must play more modulatory and subtle roles than those activated by the key biogenic amines. As such, alterations in their function may be more difficult to assess, particularly when ‘disease’ has frequently been considered in the context of animal models that have less than perfect correlation with human disease states. Furthermore, despite many years of effort, endogenously produced ligands that activate more than 100 of the non‐olfactory GPCRs remain unidentified or, at least, suggested endogenous ligands for many of these remain to be broadly accepted and agreed on by the research community. Such GPCRs are defined as ‘orphans’ (Alexander *et al.,* 2015a). This is further complicated by frequent lack of reproducibility between reports. For example, although the ‘orphan’ receptor GPR17 has been reported to respond to uracil nucleotides and cysteinyl leukotrienes (Ciana *et al.,* 2006), this has been disputed by others (Qi *et al.,* 2013; Simon *et al.,* 2017).

**Figure 1:**
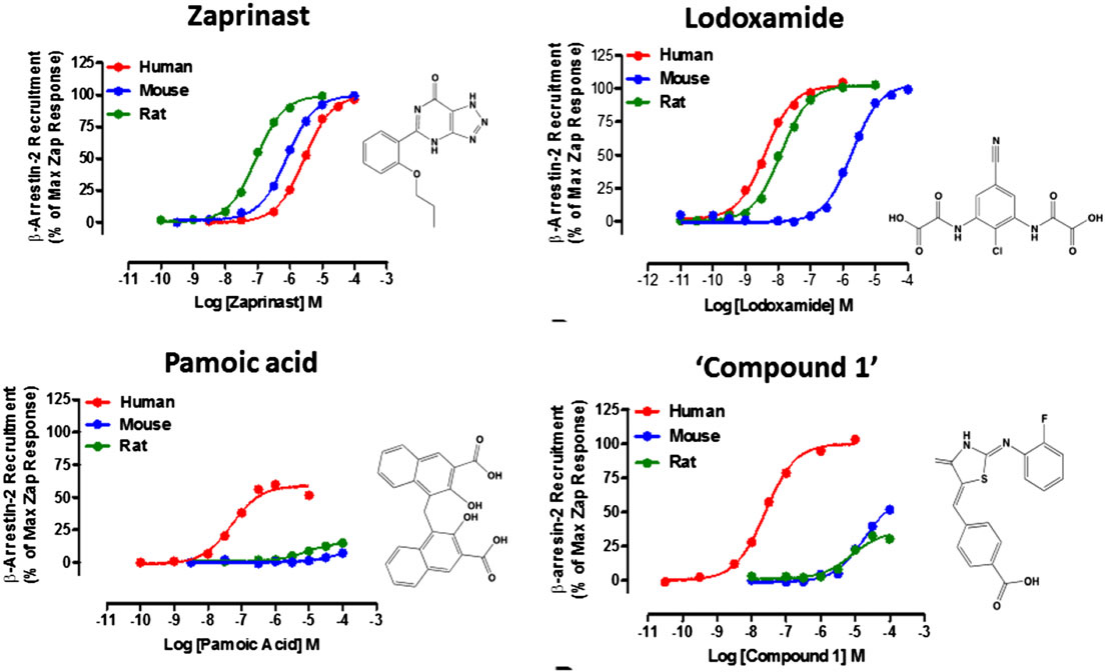
Many GPR35 agonists display markedly different potency at human and rodent orthologues. Representative concentration–response curves for four ligands with agonist potency at GPR35 are shown. All experiments reflect ligand‐induced interactions between transiently co‐expressed forms of β‐arrestin‐2 and the indicated species orthologue of GPR35. For human GPR35, the short isoform (GPR35a) was used, which corresponds to the single form of GPR35 expressed by rat and mouse. Zaprinast displays modest but similar potency at each orthologue, whilst each of lodoxamide, pamoic acid and ‘compound 1’ (4‐{(Z)‐[(2Z)‐2‐(2‐fluorobenzylidene)‐4‐oxo‐1,3‐thiazolidin‐5‐ylidene]methyl}benzoic acid) are markedly more potent at human GPR35 than at mouse GPR35 and, as such, are inappropriate to explore functions of the mouse orthologue. Data are adapted from Jenkins *et al.,* 2010, Neetoo‐Isseljee *et al.,* 2013 and Mackenzie *et al.,* 2014.

An additional reason for the current lack of small molecule medicines that target certain GPCRs is that in a number of examples of GPCRs that are activated by large peptide hormones, medicines to treat disease are based on the activating peptide itself, or on ways to prevent degradation of the peptide. This often reflects a broad ranging lack of success to date in identifying tractable starting points for small molecule activators of these GPCRs rather than a lack of confidence that stimulating the GPCR in question with such a ligand would have clinical benefit. Indeed, in the case of the http://www.guidetopharmacology.org/GRAC/ObjectDisplayForward?objectId=249, peptides related to http://www.guidetopharmacology.org/GRAC/LigandDisplayForward?ligandId=5194 or inhibitors of http://www.guidetopharmacology.org/GRAC/ObjectDisplayForward?objectId=1612 **,** the enzyme that degrades GLP‐1, are already effective therapeutic agents. There are also examples where clinical proof‐of‐concept has been produced but side effects or toxicity of the ligand tested has prevented its further development. A recent example was the withdrawal from phase III clinical trials of the http://www.guidetopharmacology.org/GRAC/ObjectDisplayForward?objectId=225&familyId=24&familyType=GPCR agonist http://www.guidetopharmacology.org/GRAC/LigandDisplayForward?ligandId=6484. This not only produced effective lowering of blood glucose levels and of glycated haemoglobin in patients with Type 2 diabetes but also induced liver toxicity by inhibiting a number of bile acid transporters (Kaku *et al.,* 2015, 2016). Such late‐stage failures in clinical studies, even if they may be related more to the specific molecule that was trialled rather than issues related more generally to the specific GPCR targeted, understandably result in considerable caution in further efforts to revisit such a target (Ghislain and Poitout, 2017; Suckow and Briscoe, 2017). However, a major issue for many molecules that have entered clinical trials, beyond potential toxicity, has been that they prove to lack efficacy in the disease state targeted. This can be the case even when various biomarker signatures provide support for appropriate target engagement for the ligand (see Pizzonero *et al.,* 2014; Namour *et al.,* 2016). As such, a more broad reaching issue which may be hindering progress towards novel medicines that function at previously non‐targeted GPCRs may be a lack of understanding of the basic biology and connectivity of systems controlled and their direct link to human disease. This can also be reflected in marked variability in the pharmacology of species orthologues of a number of poorly studied GPCRs that limits understanding of the function of such receptors in animal models (Figure 1).

I addressed a number of these issues within a Symposium on “Non‐traditional/Orphan GPCRs as therapeutic targets” held during the Pharmacology 2016 meeting in London, and the current review centres on topics considered in that presentation.

## GPCR de‐orphanization and the development of medicines

Particularly within the pharmaceutical industry, during the 1990s and 2000s, there was a drive to identify cognate ligands for many previously ‘orphan’ GPCRs. This reflected the widely held view that many more GPCRs would be defined as therapeutic targets if a much clearer understanding could be obtained of the endogenously produced ligands that activate them. This was further promoted if genetic elimination of the receptor orthologue in mice generated a distinct and clearly observed phenotype (Wise *et al.,* 2004; Civelli *et al.,* 2013). Some clear successes have emerged from this strategy. However, as with other medicine development programmes, the period between project initiation and drug agency approval has been extensive. An outstanding example has been identification of a pair of orphan GPCRs, now designated http://www.guidetopharmacology.org/GRAC/ObjectDisplayForward?objectId=321 and http://www.guidetopharmacology.org/GRAC/ObjectDisplayForward?objectId=322, as the partners for the neuropeptides http://www.guidetopharmacology.org/GRAC/LigandDisplayForward?ligandId=1697 and http://www.guidetopharmacology.org/GRAC/LigandDisplayForward?ligandId=1699. Although initially suggested to play important roles in stimulation of appetite and to affect energy metabolism, which suggested potential as therapeutic targets in obesity and control of weight gain, further studies demonstrated a key role for the orexins in the stabilization of wakefulness. Linked to the identification of inactivating mutations associated with narcolepsy within the OX_2_ receptor in dogs, this provided the drive to target these receptors in sleep disorders. While either selective OX_2_ or dual OX_1_/OX_2_ agonists could, therefore, be considered as potential treatments for human narcolepsy, little progress appears to have been made to date in efforts to identify low MW agonists of these receptors. However, low MW OX receptor antagonists were readily identified, and subsequently, this led to the development of http://www.guidetopharmacology.org/GRAC/LigandDisplayForward?ligandId=2890, a dual OX_1_/OX_2_ antagonist, which has been approved for treatment of insomnia (Norman and Anderson, 2016).

Other effective de‐orphanization efforts have not (yet) resulted in the development and approval of novel medicines, despite clear validation of the target GPCR and enormous commitment. One of the most effective means to regulate favourably blood lipid levels and composition is treatment with http://www.guidetopharmacology.org/GRAC/LigandDisplayForward?ligandId=1588 (also known as nicotinic acid) (Gille *et al.,* 2008). Niacin acts as an anti‐lipolytic agent at white fat cells and, therefore, reduces entry of stored lipids into the bloodstream. Moreover, early studies had indicated that it must do so *via* activation of a G_i_‐coupled GPCR. Identification of a receptor or receptors for niacin thus focused on a group of orphan GPCRs expressed in adipocytes. Following selection of 10 such orphan GPCRs Wise *et al*. (2003) screened each of these and demonstrated that niacin was able to increase binding of [^35^S]GTPγS in membranes prepared from cells transfected to express the receptor HM74. Two other closely related receptors HM74a and GPR81 were also able to allow a high concentration of niacin to enhance binding of [^35^S]GTPγS. Further studies showed the potency of niacin to be markedly higher at HM74a compared to HM74, with only very low potency observed at GPR81 (Wise *et al.,* 2003). IUPHAR consolidation and rationalization of nomenclature have resulted in HM74a now being designated http://www.guidetopharmacology.org/GRAC/ObjectDisplayForward?objectId=312 (Offermanns *et al.,* 2011). A marked side‐effect of treatment with niacin is the development of cutaneous vasodilatation, or flushing, on the chest and face. This markedly affects patient compliance in maintaining the treatment. As it was suggested that the flushing effects of niacin treatment might be separable from the lipid‐modifying effects, because the beneficial effects appeared to be G protein‐mediated whilst flushing appeared to be arrestin‐dependent, then efforts to identify and develop G protein‐‘biased’ agonists at this receptor have been made (Shen *et al.,* 2010; Kim *et al.,* 2015). However, these have not translated into clinical trials, and currently, although much discussed and studied, no GPCR ligand developed specifically with the concept that signalling ‘bias’ might provide distinct clinical benefits compared to non‐biased ligands at the same receptor has yet received regulatory approval. However, http://www.guidetopharmacology.org/GRAC/LigandDisplayForward?ligandId=7334, a somewhat G protein‐biased agonist at the http://www.guidetopharmacology.org/GRAC/ObjectDisplayForward?objectId=319, is currently undergoing clinical trials for pain relief (Viscusi *et al.,* 2016).

## Understanding the function of GPCRs that are not currently in the spotlight

GPCRs for biogenic amines, and for other transmitters such as acetylcholine, have been and remain the most studied family members. In part, this reflects a cycle of productivity. As among the first cloned members of the GPCR superfamily, they were the first to be expressed in heterologous systems and, apart from the photon receptor rhodopsin, the first for which atomic level structures became available. Moreover, appreciation of the key roles played by these receptors resulted in the development of a wide diversity of ligands able to either activate or antagonize their function. This was frequently associated with detailed ligand structure–activity relationships. As availability and use of tool compounds is central to all aspects of pharmacology, then this has further consolidated research focus on a modest number of the GPCR superfamily. Moreover, because modes of activation and regulation of different members of the GPCR family are broadly similar, much that has been learned from studies on rhodopsin and β‐adrenoceptors has general relevance to less‐studied receptors. Despite this, subtle variations are likely to be integral to specific functions of different family members, and therefore, it is unwise to assume, as many do, that the most studied GPCRs provide appropriate answers for drug design and development at less studied GPCRs. As in many other areas of target validation, knowledge of GPCR expression patterns, information on phenotypes associated with knock‐down of expression or inactivation of the corresponding gene and either receptor variant or single nucleotide polymorphisms linked to human disease are vital to help prioritize ‘orphan’ or poorly characterized GPCRs as potential therapeutic targets worthy of detailed study.

## GPR35 and FFA2 receptors: two incompletely understood metabolite‐sensing GPCRs

In recent years, the contribution of foodstuffs, and metabolites derived from them, as GPCR activators and homeostatic regulators of metabolic and immune function has become abundantly clear (see Blad *et al.,* 2012, Alvarez‐Curto and Milligan, 2016, Tan *et al.,* 2017 for review). Despite this, a number of the GPCRs that respond to such ligands and appear to play key roles in such effects remain poorly or incompletely characterized. Herein, I will focus on two such GPCRs, the ‘orphan’ receptor http://www.guidetopharmacology.org/GRAC/ObjectDisplayForward?objectId=102&familyId=16&familyType=GPCR (Divorty *et al.,* 2015; Mackenzie and Milligan, 2017) and the http://www.guidetopharmacology.org/GRAC/ObjectDisplayForward?objectId=226&familyId=24&familyType=GPCR (FFA2) (Bolognini *et al.,* 2016a; Milligan *et al.,* 2017a, b), a receptor responsive to short‐chain fatty acids (SCFAs), and suggest why this remains so. This is despite an FFA2 receptor antagonist already having being assessed in phase II clinical trials.

## FFA2 receptors: opportunities and challenges

Previously designated GPR43 (Sawzdargo *et al.,* 1997) and de‐orphanized in 2003 as a receptor for http://www.guidetopharmacology.org/GRAC/LigandDisplayForward?ligandId=1062 and other SCFAs (Brown *et al.,* 2003), the FFA2 receptor has been studied extensively (Bolognini *et al.,* 2016a; Milligan *et al.,* 2017a). In significant part, this reflects the wide‐ranging roles suggested for SCFAs. These metabolic products are generated by the microbiota mainly in the lower gut by fermentation of non‐digestible carbohydrates. The integral role that the microbiota plays in health and disease is a topic of ever expanding interest. However, for pharmacologists, studying FFA2 receptors presents many challenges. Although it is clear that SCFAs are indeed the endogenous activators of this GPCR, concentrations that induce responses are in the high μM to low mM range. Moreover, a second receptor, free fatty acid receptor 3 (FFA3), responds to the same group of SCFAs (Bolognini *et al.,* 2016a). Although there is a distinct structure–activity relationship for the SCFAs between the two receptors, there is insufficient separation to use any individual SCFA in an *ex vivo* or *in vivo* context to specify with confidence the receptor involved in a response (Bolognini *et al.,* 2016a; Milligan *et al.,* 2017b). This is exacerbated because potencies of the SCFAs differ between human and rodent orthologues of these receptors. This feature has been attributed to differences in the potential to form pairs of extracellular ‘ionic locks’ between conserved arginine residues that form the core of the orthosteric binding pockets and negatively charged residues within the second extracellular loop (Hudson *et al.,* 2012a). Further issues stem from the close linkage of the genes encoding FFA2 and FFA3 receptors at chromosome 19q13.1 in human and 7 A3 in mouse. Indeed, in at least one reported example, knockout of FFA2 receptors in mouse resulted in compensatory up‐regulation of levels of FFA3 receptor mRNA in white adipose tissue (Bjursell *et al.,* 2011), complicating interpretation of the study outcomes.

The greatest limitation for pharmacological studies, however, has been the paucity of selective and pan‐species active FFA2 or FFA3 receptor ligands. Although at least two series of FFA2 receptor selective, orthosteric agonist ligands have been reported in the patent literature (Milligan *et al.,* 2017b), exemplars from these series have, to date, been employed sparingly in peer‐reviewed publications. This is likely to reflect a combination of a remaining caution of certain pharmaceutical companies to provide such ligands to the academic community, alongside the often poor links between academic pharmacologists and medicinal chemists, which could and should allow synthesis and study of patented ligands for basic underpinning research. There are, of course, exceptions to this. 3‐Benzyl‐4‐(cyclopropyl‐(4‐(2,5‐dichlorophenyl)thiazol‐2‐yl)amino)‐4‐oxobutanoic acid (http://www.guidetopharmacology.org/GRAC/LigandDisplayForward?ligandId=6488 in Hudson *et al.,* 2013), initially exemplified in a patent from Euroscreen (now Ogeda SA), was synthesized and used to show the capacity of FFA2 receptors to act both as anti‐lipolytic receptors in model adipocytes of both human and murine origin (Hudson *et al.,* 2013) and to promote release of the incretin GLP‐1 from the murine enteroendocrine cell line STC‐1 (Hudson *et al.,* 2013). Furthermore, (2S, R5)‐5‐(2‐chlorophenyl)‐1–1(2′‐methoxy‐[1,1′‐biphenyl]‐4carbonyl)pyrrolidine‐2‐carboxylic acid (‘compound 1’ in Forbes *et al.,* 2015), also described initially in a patent from Euroscreen (Milligan *et al.,* 2017a, b), has been used to show a role of FFA2 receptors in inhibiting intestinal functions and suppression of food intake *via* http://www.guidetopharmacology.org/GRAC/FamilyIntroductionForward?familyId=46‐mediated pathways in mice. These highlight means by which pharmacologists may be able to access patented and, therefore, publically described, GPCR‐active ligands that are not available from commercial sources.

**Figure 2:**
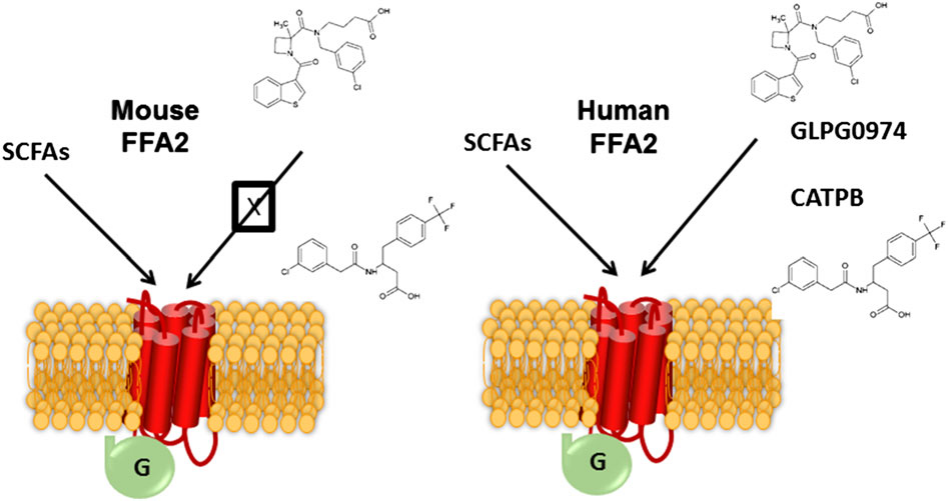
Mouse and human orthologues of FFA2 receptors display extreme differences in antagonist ligand pharmacology. For both mouse and human FFA2 receptors, short‐chain fatty acids induce activation of a panoply of signals *via* different members of the family of heterotrimeric G proteins (see Figure 3). However, antagonists from two reported chemical series, exemplified by GLPG0974 and CATPB, display no significant affinity at mouse FFA2 receptors, although they are high‐affinity, orthosteric antagonists at the human FFA2 receptor.

In many settings, antagonist ligands can offer wider opportunities to define specific roles for GPCRs. At least three separate classes of FFA2 receptor antagonists have been described (Milligan *et al.,* 2017a, b). However, although exemplars from each series are able to block agonist actions at human FFA2 receptors, none of them display significant affinity at rodent orthologues of this receptor (Milligan *et al.,* 2017a, b) (Figure 2). Thus, whilst http://www.guidetopharmacology.org/GRAC/LigandDisplayForward?ligandId=6487 was able to block the anti‐lipolytic effect of ‘compound 1’ in human SW872 adipocytes, it was unable to do so in murine 3T3‐L1 cells (Hudson *et al.,* 2013). Equally, whilst http://www.guidetopharmacology.org/GRAC/LigandDisplayForward?ligandId=8417 is able to block http://www.guidetopharmacology.org/GRAC/LigandDisplayForward?ligandId=1058‐induced chemotaxis of human neutrophils (Pizzonero *et al.,* 2014), this compound is all but inactive at rodent orthologues of FFA2 receptors (Pizzonero *et al.,* 2014; Sergeev *et al.,* 2016) and unable to bind to mouse FFA2 receptors with significant affinity (Sergeev *et al.,* 2016).

Despite this challenge, Galapagos NV developed GLPG0974 for clinical studies using a biomarker for target engagement in human blood, based on the ability of GLPG0974 to prevent acetate‐induced up‐regulation of the ‘activation epitope’ of the integrin http://www.guidetopharmacology.org/GRAC/FamilyDisplayForward?familyId=760#2452 (Pizzonero *et al.,* 2014). Outcomes of the subsequent early‐stage clinical studies have been reported by Namour *et al*. (2016), but, sadly, phase II trials failed to demonstrate efficacy of GLPG0974 in patients with ulcerative colitis and these trials, therefore, were terminated. Although a series of rodent disease‐model studies might have been instructive, this was not practical for GLPG0974 due to the lack of affinity of GLPG0974 at rodent orthologues of FFA2 (Figure 2). Interestingly Hudson *et al*. (2012b) noted that bovine FFA2 receptors responded optimally to fatty acids of somewhat longer chain length than do the human or rodent forms. Based on this, and sequence alignments, they were able to generate a mutationally modified ‘designer receptor exclusively activated by designer drug’ (DREADD) form of human FFA2 receptor that no longer responded to the SCFAs acetate or propionate but was activated instead by a number of non‐endogenous ligands, including sorbic acid (Hudson *et al.,* 2012b). Importantly, the mutations introduced to develop the DREADD variant do not interfere with binding of FFA2 receptor antagonists from either of the described high‐affinity series. Notably, Bolognini *et al*. (2016a) have highlighted that production of transgenic mice in which either a humanized form of FFA2 receptors or a humanized form of the DREADD FFA2 receptor is expressed in place of the mouse orthologue has been achieved. Although pharmacological studies on these animals, and tissues derived from them, have not yet been reported, it is hoped that such studies will shed new light on the patho‐physiological roles of FFA2 receptors and allow the human‐specific FFA2 receptor antagonists to block effects of orthosteric agonists and define potential ‘on‐target’ versus ‘off‐target’ effects of SCFAs in tissues derived from these animals, and even potentially directly in *in vivo* studies.

## FFA2 receptors: orthosteric versus allosteric agonists

**Figure 3:**
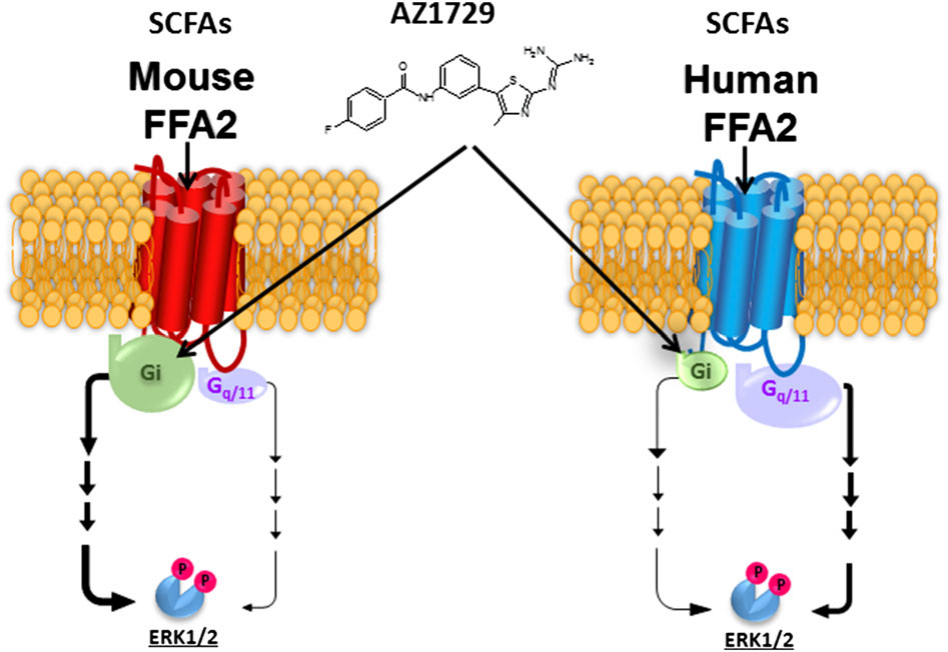
Species orthologues of FFA2 receptors display G protein selectivity. SCFAs promote phosphorylation and activation of ERK1/2 MAP kinases in HEK293 cells transfected to express either human or mouse FFA2. However, combinations of studies with the G protein inhibitors *Pertussis* toxin (G_i_) and FR900359 (G_q_/G_11_) and the G_i_‐biased FFA2 agonist AZ1729 demonstrate that while activation of the human orthologue transmits this signal largely *via* G_q_/G_11_, for the mouse orthologue the signal is transmitted largely *via* G_i_. The indicated size of the noted G protein subtypes illustrates the relative contribution of each to SCFA‐mediated activation of ERK1/2 MAP kinases. See text and Bolognini *et al*. (2016b) for further details.

It is often stated that there must be evolutionary pressure to maintain orthosteric ligand‐binding pockets in GPCRs to avoid loss of function. By contrast, an argument can be made that this should be less intense for allosteric sites that recognize synthetic compounds to which the receptor has never naturally been exposed. This does not mean, however, that allosteric ligands are intrinsically species orthologue selective. In the case of FFA2 receptors, two key allosteric agonists have been described. http://www.guidetopharmacology.org/GRAC/LigandDisplayForward?ligandId=3420 (Lee *et al.,* 2008; Smith *et al.,* 2011; Grundmann *et al.,* 2016) and AZ1729 (Bolognini *et al.,* 2016b) are each able to directly activate both human and rodent forms of FFA2 receptors and do so *via* one or more allosteric sites. AZ1729 is potentially of particular interest to pharmacologists as it acts as a highly ‘biased’ agonist. While SCFAs induce conformations of FFA2 receptors able to interact with both G_i_ and G_q_‐family G proteins, AZ1729 is able to promote signalling only *via* G_i_‐mediated mechanisms (Bolognini *et al.,* 2016b). As such, although AZ1729 is able to induce anti‐lipolytic effects in adipocytes *via* FFA2 receptors, in a *Pertussis* toxin‐sensitive and, therefore, G_i_‐mediated manner, it is unable to promote release of GLP‐1 from mouse colonic crypts. By contrast SCFAs do so, and in a manner that is instead blocked by the selective G_q_/G_11_ inhibitor http://www.guidetopharmacology.org/GRAC/LigandDisplayForward?ligandId=9336 (Bolognini *et al.,* 2016b). AZ1729 has, therefore, been posited to provide a means to determine the contribution of G_i_‐G proteins to downstream signal transduction processes that may coalesce from multiple signalling pathways. The best example to date of such a use of AZ1729 has been to define the contribution of G_i_‐family G proteins to the regulation of http://www.guidetopharmacology.org/GRAC/FamilyDisplayForward?familyId=514 phosphorylation following expression of species orthologues of FFA2 receptors in HEK293 cells. For human FFA2 receptors in naïve cells, the SCFA propionate produces a very robust stimulation of ERK1/2 phosphorylation, whereas AZ1729 produces only very limited activation. Following treatment with *Pertussis* toxin to inactivate G_i_‐proteins, the signal induced by AZ1729 was abolished, whereas responses to propionate remained robust. By contrast after treatment of cells with the G_q_/G_11_ inhibitor FR900359, the modest response to AZ1729 was maintained while the response to propionate was now as limited, as for AZ1729 (Bolognini *et al.,* 2016b). This implies that the key transducers of this effect are the G_q_/G_11_‐family proteins (Figure 3). A completely different pattern was observed, however, for the mouse FFA2 receptor. Here, equivalent experiments to those described above showed that activation of ERK1/2 phosphorylation *via* this orthologue is transduced almost entirely by the G_i_‐family G proteins (Figure 3). Thus, although focus is routinely on differences in ligand pharmacology at receptor species orthologues, these results show that selectivity of GPCR‐G protein coupling, as recently considered by Flock *et al*. (2017), can be observed, presumably due to differences on the intracellular, G protein‐contact surface of the receptor. Understanding the molecular basis for such effects and how they may shape different physiological outcomes in rodent versus human tissues may be as important in terms of smoothing the path of medicine development as the more widely (and wildly) championed concept of ‘ligand bias’.

The characterization and use of AZ1729 (Bolognini *et al.,* 2016b) is an interesting example of ‘Open Innovation’ policies, now being adopted more widely by pharmaceutical companies. Here, both early stage, far from optimized, ligands but also in some cases clinically trialled molecules, are being made available to academic researchers to promote better understanding of underpinning biology and, therefore, potential target validation, as part of ‘pre‐competitive’ programmes of research. In the example of AZ1729, this molecule was scored simply as a ‘hit’ in a high‐throughput screen conducted internally by AstraZeneca, and the details of its mode of action were defined subsequently within an academic–industrial collaboration.

## GPR35: opportunities and challenges

**Figure 4:**
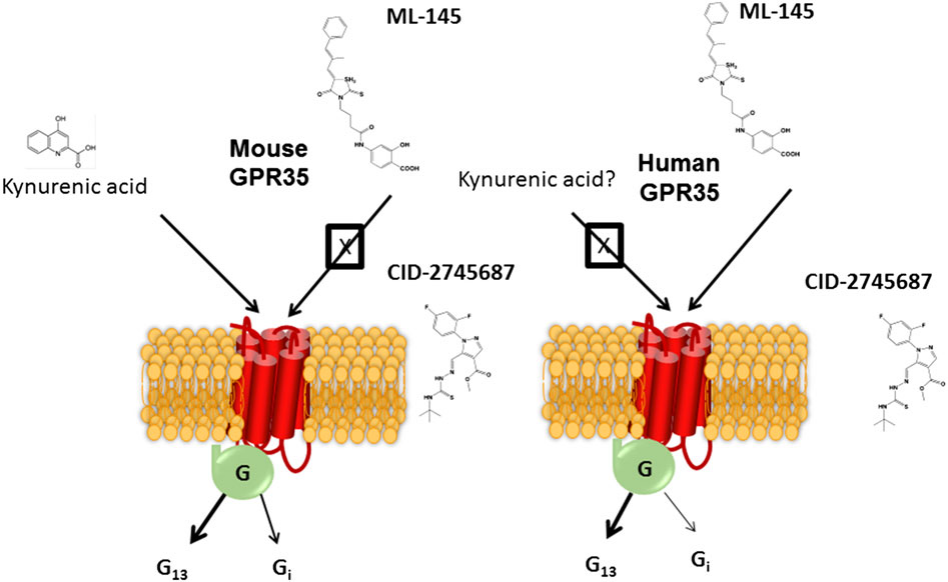
Mouse and human orthologues of GPR35 display marked differences in antagonist ligand pharmacology. Although the tryptophan metabolite kynurenic acid is able to activate GPR35, it is more potent at the mouse orthologue that at either splice variant of the human orthologue. This has raised questions of its effectiveness as an agonist at GPR35 in human. Exemplars from two chemical series, illustrated as ML‐145 and CID‐27455687, have been shown to be high‐potency antagonists of human GPR35. However, at least in transfected cell systems, neither appears able to block agonist actions at mouse GPR35 (Jenkins *et al.,* 2012). However, in certain reports, these ligands have been reported to block effects at GPR35 in rodent cells and tissue (see text for details). These dichotomies remain to be understood, but improved pharmacological study is likely to do so. G protein‐mediated signalling resulting from activation GPR35 in different cells and tissues can involve both Gα_13_ and *Pertussis* toxin‐sensitive members of the G_i_‐subfamily.

GPR35 remains an ‘orphan’ receptor although both kynurenic acid (Wang *et al.,* 2006) and the chemokine http://www.guidetopharmacology.org/GRAC/LigandDisplayForward?ligandId=6479 (Maravillas‐Montero *et al.,* 2015) have been proposed as endogenous ligands. CXCL17 has been championed as a ligand in a single publication and, therefore, requires independent support from other studies and approaches. However, whilst Maravillas‐Montero *et al*. (2015) highlighted structurally conserved features between GPR35 and highly characterized chemokine receptors, including http://www.guidetopharmacology.org/GRAC/ObjectDisplayForward?objectId=80 and http://www.guidetopharmacology.org/GRAC/ObjectDisplayForward?objectId=71, a recent re‐stratification of class A GPCRs based on homology within core ligand‐binding domains that effectively clustered CXCR7 and CXCR4 and other *bona fide* receptors for chemokines placed GPR35 on a completely separate branch (Ngo *et al.,* 2017). In the case of http://www.guidetopharmacology.org/GRAC/LigandDisplayForward?ligandId=2918, although it is clear from many publications that this molecule, which is produced endogenously by metabolism of the amino acid tryptophan, can activate GPR35 if provided at sufficiently high concentrations, marked variation in reported potency of kynurenic acid at species orthologues of the receptor (Milligan, 2011) (Figure 4) has hindered acceptance of this metabolite as the true endogenous agonist. Indeed, potency of kynurenic acid is particularly low at human GPR35 (Jenkins *et al.,* 2011), and this may exclude a role for kynurenic acid at human GPR35 (Figure 4).

The potential of targeting GPR35 in a therapeutic context is supported in part by genetic associations that have suggested roles in diseases including ulcerative colitis and primary sclerosing cholangitis (Ellinghaus *et al.,* 2013; Yang *et al.,* 2015), and GPR35 is highly expressed in regions of, particularly, the lower gut and colon and by various white cell groups, including numerous dendritic cell and monocyte populations. Potential roles for GPR35 in the regulation of pain have also been supported (Resta *et al.,* 2016; Khan and He, 2017; Mackenzie and Milligan, 2017) as the receptor in also expressed regionally in both the central and peripheral nervous systems.

## GPR35: ligand pharmacology and species variation

Sequence variation between human, rat and mouse orthologues of GPR35 is substantial (Milligan, 2011). Unsurprisingly, therefore, this is manifest in marked variation in agonist ligand pharmacology. Like FFA2 receptors, this is also the case for antagonist ligands for GPR35 (Figure 4) and creates major challenges for pharmacological definition of receptor functions. Two series of relatively high affinity GPR35 antagonists have been reported (Heynen‐Genel *et al.,* 2010, 2011), but, although effective blockers of human GPR35 (Jenkins *et al.,* 2012), at least following heterologous expression of rat and mouse GPR35 into HEK293 cells, neither of the currently commercially available antagonist ligands, CID‐2745687 and ML‐145 (Figure 4), are able to block agonist effects at these orthologues (Jenkins *et al.,* 2012). It is, therefore, somewhat confusing that ML‐145 has been reported to partially block effects of the GPR35 activator http://www.guidetopharmacology.org/GRAC/LigandDisplayForward?ligandId=2919 in rat CA1 hippocampal neurons (Alkondon *et al.,* 2015). Clearly, it is possible that in natively expressing systems, co‐expressed proteins that are not present in HEK293 cells may alter the pharmacology of a GPCR, as is known for the small group of single transmembrane domain receptor activity modulating proteins (Gingell *et al.,* 2016; Hay *et al.,* 2016). However, the structure of ML‐145 contains a central rhodanine fragment, often found in so‐called PAINS (pan‐assay interference compounds) (Everett, 2015). Thus, it is likely that when used in *ex vivo* preparations, ML‐145 will have a range of ‘off‐target’ effects that are unappreciated. The limited interaction of many academic pharmacologists with drug discovery experts and medicinal chemists based in either academia or the pharmaceutical industry raises the real danger of lack of appreciation of such issues, and an over‐optimistic hope that the commercial availability of various compounds, and their described ‘selectivity’ on provider websites, is valid. Thus, without support from each of orthogonal assays, the parallel use of tissue from ‘knockout’ animals, or the use of as wide a pharmacology as available for a poorly characterized GPCR, then conclusions as to its physiological functions should only be considered as interim. Equally, despite the results of Jenkins *et al*. (2012) indicating a lack of action of CID‐2745687 at mouse GPR35, CID‐2745687 had previously been reported to inhibit agonist‐induced interactions between mouse GPR35 and a form of β‐arrestin 2 tagged with a fluorescent protein in HEK293 cells (Zhao *et al.,* 2010) and, more recently, to inhibit the ability of kynurenic acid to affect forskolin‐mediated regulation of cAMP levels in mouse astrocytes (Berlinguer‐Palmini *et al.,* 2013) and to block effects of a GPR35 agonist in young adult mouse colon epithelial cells (Tsukahara *et al.,* 2017). These results are clearly incompatible with the findings of Jenkins *et al*. (2012), assuming that CID‐2745687 was actually acting *via* GPR35 in these studies. However, in a more positive context, in isolated human saphenous vein smooth muscle cells, GPR35 agonist‐induced cell lengthening and cell migration were both blocked effectively by concentrations of both CID‐2745687 and ML‐145 consistent with reports on the affinity of these ligands at human GPR35, as defined from *in vitro* studies (McCallum *et al.,* 2015), as was proliferation of human saphenous vein endothelial cells (McCallum *et al.,* 2015).

## GPR35: signalling mechanisms

A further challenging feature of GPR35 is the mechanism(s) this receptor employs for signal transduction. While signalling in a number of, particularly, neuronal preparations appears to be mediated *via* one or more *Pertussis* toxin‐sensitive G proteins (Figure 4) and can regulate levels of cAMP (Mackenzie and Milligan, 2017), in many other cases, it appears that key G protein‐mediated effects proceed *via* the poorly studied G protein Gα_13_ (Jenkins *et al.,* 2010, 2011). This has been assessed in experiments measuring agonist‐induced binding of [^35^S]GTP[S] to an introduced epitope‐tagged form of Gα_13_ (Jenkins *et al.,* 2010) and in assays employing a chimeric Gα_q_/Gα_13_ G protein that allows receptor interaction with Gα_13_ to be converted into elevation of intracellular Ca^2+^ levels (Jenkins *et al.,* 2011). Moreover, in human saphenous vein smooth muscle cells, GPR35 agonist‐induced cell migration is inhibited by each of two mechanistically distinct Rho‐kinase inhibitors (McCallum *et al.,* 2015). Although activation of Gα_13_ does not directly regulate levels of secondary messengers, it does regulate Rho‐guanine nucleotide exchange factors. In some of the earliest detailed studies, chimeric G proteins based on the backbone of the α subunit of the Saccharomyces cerevisiae G protein Gpa1 (Jenkins *et al.,* 2010) or the mammalian G protein G_q_ (Jenkins *et al.,* 2011) were used to show effective coupling of GPR35 to Gα_13_, and that this was the case for both human and rat GPR35 (Jenkins *et al.,* 2011). Although G_13_ is often viewed as being functionally equivalent to the related G protein G_12_, this is unlikely to be the case as both genes have been preserved across species, and mouse gene ‘knockout’ studies confirm that they are non‐redundant, with lack of only Gα_13_ resulting in early embryonic lethality linked to a lack of angiogenesis (Worzfeld *et al.,* 2008). Indeed, in parallel studies, whilst a G_q_‐Gα_13_ chimeric G protein was able to link both human and rat GPR35 to elevation of intracellular Ca^2+^ levels, an equivalent G_q_‐Gα_12_ chimera was only very weakly active, whilst full length Gα_q_ was entirely without function (Jenkins *et al.,* 2011).

In part, because selective activation of Gα_13_ is highly challenging to assess in any high‐throughput assay format, efforts to identify novel agonists of GPR35 have focussed on either the use of ‘dynamic mass redistribution’ studies, performed almost exclusively using the human HT29 adenocarcinoma cell line (e.g. Wei *et al.,* 2017) that expresses GPR35 endogenously but where the mode of signalling is undefined, or various assay formats that measure induced translocation and interaction with the receptor, of forms of β‐arrestin (Zhao *et al.,* 2010; Jenkins *et al.,* 2011; Funke *et al.,* 2013; Neetoo‐Isseljee *et al.,* 2013; MacKenzie *et al.,* 2014). Where tested, these have confirmed that many GPR35 agonists display marked species‐dependent variation in potency (Figure 1), but a number of molecules, including http://www.guidetopharmacology.org/GRAC/LigandDisplayForward?ligandId=2920, lodoxamide and ligands based on 8‐amido‐chromen‐4‐one‐2‐carboxylic acid, that display high potency at one or more species orthologue (usually human) have been identified (Divorty *et al.,* 2015). However, as discussed earlier for the antagonist ML‐145, a number of these ligands are appreciated to have other molecular targets, with pamoic acid identified initially as a GPR35 agonist because pamoate was the common feature in a number of compound preparations that appeared as active at GPR35 but without other obvious common chemical signatures (Jenkins *et al.,* 2010, Zhao *et al.,* 2010, see Neubig, 2010 for review). This is akin to the de‐orphanization of FFA2 receptors (Brown *et al.,* 2003), where the common feature of apparent hits was that they were all tested initially as acetate salts. The focus on using β‐arrestin‐based assays in identification of ligands may result in the prioritization of β‐arrestin‐‘biased’ agonists, but this issue has not been assessed directly, potentially once more due to the challenges of developing robust G protein‐dependent assays for GPR35. To date, only one publication has described the development and use of a radiolabelled ligand to identify and characterize the pharmacology of GPR35. [^3^H]PSB‐13253 (6‐bromo‐8‐(4‐methoxybenzamido)‐4‐oxo‐4H‐chromene‐2‐carboxylic acid) (Thimm *et al.,* 2013) displayed high affinity (K_d_ = 5 nM at human GPR35). As the parent compound is a markedly selective agonist for human GPR35 compared to rat or mouse (Funke *et al.,* 2013) then, although not explored extensively by Thimm *et al*. (2013), it is unlikely to be useful to study rodent orthologues of GPR35. Moreover, all the studies reported by Thimm *et al*. (2013) were performed using membrane preparations from CHO cells expressing very high levels of human GPR35, estimated to be in the region of 12 pmol·mg^−1^ membrane protein. It remains to be established if [^3^H]PSB‐13253 can be used to detect and study the pharmacological profile of human GPR35 in cells or tissues that express the receptor endogenously. As with FFA2 receptors, it may be that transgenic mice expressing a humanized version of GPR35 could provide an effective means of helping to better define potential therapeutic roles for ligands at GPR35. However, given the relative dissimilarity of mouse and human GPR35 sequences then, as noted above for FFA2 receptors, despite the evidence to date that suggests that both human and rodent forms of GPR35 show marked selectivity in coupling to Gα_13_, it is possible that G protein engagement patterns will vary between orthologues and result in distinct downstream signalling outcomes.

A further challenge on how to interpret data from GPR35 studies in animal models for its likely effects on the therapeutic opportunities offered by GPR35 in the treatment of human disease is that, although both rat and mouse express a single form of the receptor, there are two forms in human. The short isoform corresponds to the rodent orthologue, but there is also an N‐terminally extended splice variant that incorporates an additional 31 amino acids into this region of the receptor (Milligan, 2011). Although there is little evidence to date of distinct pharmacology between the human short (GPR35a) and long (GPR35b) variants, it may be that these will differ in details of localization as is the case, for example, for the GABA_B1a_ and GABA_B1b_ isoforms of the http://www.guidetopharmacology.org/GRAC/ObjectDisplayForward?objectId=240.

## Conclusions

It can be argued that the key focus to date on GPCRs that are activated by biogenic amines simply reflects the high medical importance and prevalence of diseases that are associated with dysregulation of these ancient regulators of a broad raft of physiological processes. However, as pharmacological studies are entirely reliant on access to high‐quality and well‐characterized tool compounds, this focus has become something of a self‐fulfilling tradition. Progress in understanding less well‐characterized GPCRs will require combinations of yet greater willingness of companies to offer access to tool compounds. It is also vital that academic pharmacologists interact much more closely with medicinal chemists in both the academic and industrial spheres to make better use of the plethora of novel ligands described within the patent literature, and for pharmacologists to be far more discerning in selecting, or further characterizing, ligands to ensure they are fit for purpose and truly display the selectivity or specificity attributed to them. With these basic tenets in place, the potential for much more convincing target validation of GPCRs that are ‘not currently in the spotlight’ will be greatly enhanced to the benefit of pharmacologists, the pharmaceutical industry and ultimately, patients.

### Nomenclature of targets and ligands

Key protein targets and ligands in this article are hyperlinked to corresponding entries in http://www.guidetopharmacology.org, the common portal for data from the IUPHAR/BPS Guide to PHARMACOLOGY (Southan *et al.,* 2016), and are permanently archived in the Concise Guide to PHARMACOLOGY 2015/16 (Alexander *et al.,* 2015a, b, c).

## Conflict of interest

The author declares no conflicts of interest.
